# Impact of Dedicated Nurse Time on Electronic Health Record (EHR)-Based Diabetes Quality Measures: A Pre–Post Observational Study

**DOI:** 10.7759/cureus.105341

**Published:** 2026-03-16

**Authors:** Bryan A Farford, Jacqueline K Hurd, Jeffery L Crick, Manisha Salinas, Christine Q Nguyen, Raphael A. O Bertasi, Dani G Zapp, Tyler M Janitz, George G. A Pujalte

**Affiliations:** 1 Family Medicine, Mayo Clinic, Jacksonville, USA; 2 Nursing, Mayo Clinic, Jacksonville, USA; 3 Epidemiology and Public Health, Center for Health Equity and Community Engagement Research, Mayo Clinic, Jacksonville, USA; 4 Internal Medicine, Advent Health, Orlando, USA

**Keywords:** data collection, diabetes, electronic health record, quality improvement, registered nurse

## Abstract

Background and objective: Despite major advances in diabetes therapies, only about 60% of U.S. patients achieve recommended glycemic targets in primary care. Inconsistent electronic health records (EHR) complicate diabetes care delivery and highlight the need for strategies that support accurate documentation and patient engagement. The objective of this project is to have 50% of patients with diabetes meet six quality measures: hemoglobin A1c, blood pressure, statin use, aspirin use, tobacco status, and urine microalbumin.

Methods: In April 2019, a registered nurse (RN) was tasked with overseeing these six diabetes quality measures among 477 patients with diabetes. Using electronic health record (EHR) reports, the RN identified patients not meeting measures and implemented interventions, including patient outreach, laboratory coordination, medication reconciliation, and documentation updates. The primary outcome was the proportion of patients meeting all six measures (“all-or-none compliance”). The clinic transitioned its electronic health record (EHR) from Cerner to Epic in October 2018, which affected quality reporting during the study period.

Results: All-or-none compliance was 40.0% during the pre-EHR transition phase, declined to 24.6% after EHR implementation (pre-RN phase), and increased to 40.6% during the RN intervention phase. At the start of the RN intervention, mean compliance was 35% and improved by an average of 45.7 patients (1.2%) per month through December 2019. RN review identified multiple deficiencies related to EHR documentation, including unreconciled medications, outdated tobacco status, missing external laboratory results, and patients no longer affiliated with the practice. Addressing these issues improved the accuracy of quality reporting and completion of required measures.

Conclusion: Diabetes often carries a complex care plan, initiated and managed in most primary care settings. It requires a multidisciplinary approach with EHR facilitating quality measurement. Dedicating an RN to oversee the quality of measures of patients with diabetes may improve the accuracy and completeness of EHRs.

## Introduction

The total cost of diabetes care in the US increased by more than 26% from 2012 to 2017, to $327 billion, and diabetes is the seventh leading cause of death [1,2]. The overall health impact on society leads to a two- to three-fold increased risk of heart attack and stroke, particularly among African and Asian Americans, Hispanic and Latino Americans, Pacific Islanders, and Native Americans [3]. Diabetes is the cause of 3% of the world’s blindness and is the leading cause of kidney failure [4,5]. Therefore, improving the quality of care for this disease requires the attention of a multidisciplinary team to deliver the highest quality of evidence-based care possible.

Throughout the past 100 years, starting with the use of insulin in 1922, considerable effort has been made to improve diabetes care through research, development of medications, and evidence-based screening and evaluation tools [6]. Additionally, the number of patients with diabetes seeking primary care is on the rise. In 2016, US family medicine physicians had over 200 million office visits for diabetes care [7]. Despite the advancements in diabetes care and the increasing number of encounters with primary care physicians (PCPs), only about 60% of patients with diabetes in the US are meeting glycemic goals (hemoglobin A1c <7) [8].

Many factors play a role in the challenges of diabetic management. For instance, patient non-adherence is a major barrier to optimal care. One systematic review of antidiabetic therapies found adherence rates between 36% and 93% [9]. Patient non-adherence may be the result of several factors, including cost of treatment, adverse effects of medication, educational barriers, patient beliefs, and access to care [10]. Time limitation is another challenge facing PCPs, as it can take more than 48 hours per year to provide appropriate care for one patient with controlled diabetes and approximately 79 hours per year to manage a patient with uncontrolled diabetes [11]. In addition to diabetes, PCPs are challenged with managing multiple chronic medical conditions, addressing acute care needs, and recommending preventive care services. These competing issues limit the amount of time that can be spent providing appropriate education and care to patients with diabetes.

Further challenges to managing diabetes include inconsistent data collection and reporting in the electronic health record (EHR). These inconsistencies can inaccurately portray a lack of quality care. For example, apparent insufficient documentation on appropriate labs, such as hemoglobin A1c or urine microalbumin levels, may be the result of embedded scanned documents or records not shared with the entire medical team. Therefore, the data collected for reporting or research purposes may be unsuitable [12]. Other complicating factors for diabetes management include the constant push toward financial incentive payment value-based programs led by the Centers for Medicare & Medicaid Services (CMS) [13]. These programs have caused physician practices to invest heavily in EHRs for improved data collection and reporting, which has not only been costly but also inconsistent in providing reliable data.

It is imperative for individual practices to operate accurate EHRs and use evidence-based quality improvement processes with a multidisciplinary team to improve the quality of care for patients with diabetes. Teams that include PCPs, nurse diabetic educators, dietitians, and community support programs can help patients reach diabetic goals, prevent major adverse effects, and improve quality of life [14]. To enhance the quality of care for patients with diabetes, a registered nurse (RN) was allotted dedicated time in a family medicine practice to assist physicians with improving quality measures for their patients.

## Materials and methods

In April 2019, an RN was recruited to assist with the management of patients with diabetes in a Northeast Florida family medicine practice. The practice has four physicians, four advanced practice RNs (APRNs), and over 9,000 patients. At the time of the study (April 1, 2019-December 31, 2019), 477 patients had a diagnosis of diabetes in their EHR. Patients in this practice were only assigned to 1 physician. APRNs partnered with physicians to co-manage a group of patients.

The RN oversaw six diabetes-related quality measures, with the majority of time dedicated to EHR chart review and reconciliation rather than direct patient care. The measures included hemoglobin A1c, blood pressure, statin use, aspirin use, tobacco status, and urine microalbumin measurement (Table 1).

**Table 1:** Measurements goals and RN engagement activities and strategies BP: blood pressure; EHR: electronic health record; HbA1c: hemoglobin A1c; LDL-C: low-density lipoprotein cholesterol; RN: registered nurse. a Documented allergy or intolerance to statin medications was counted as meeting this measurement. b Documented allergy to aspirin was counted as meeting this measurement.

Measurement	Achievement goal	RN engagement strategies
HbA_1c_ control	Percentage of patients 18-75 years of age with a diagnosis of diabetes with most recent HbA_1c_ result <8% during the study period	Placing orders for labs and follow-up visits Gathering outside labs and records and updating the EHR
BP control	Percentage of patients 18-75 years of age with a diagnosis of diabetes with most recent BP measurement during the study period <140/90	Arranging a BP check within the clinic Arranging appropriate follow-up with physician if needed
LDL-C control/statin use	Percentage of patients 18-75 years of age with a diagnosis of diabetes with most recent LDL-C (within the last 5 years) of £70 mg/dL or prescription for statin to treat elevated cholesterol levels^a^	Briefly educating patients on the importance of statin use Arranging follow-up labs and physician visits when needed Working with the physician to prescribe statin if needed Gathering outside labs and records and updating the EHR Documenting intolerance or allergy to statins
Aspirin or antithrombotic use	Percentage of patients 18-75 years of age with a diagnosis of diabetes and comorbidity of intravascular disease with an active order for aspirin or antithrombotic medication^b^	Briefly educating patients on the importance of aspirin therapy Having the patient start aspirin therapy when appropriate, Updating the EHR to reflect aspirin use or allergy
Tobacco use	Percentage of patients 18-75 years of age with a diagnosis of diabetes documented to be tobacco free during the study period	Updating tobacco use in EHR Briefly counseling patient on tobacco cessation when appropriate
Urine microalbumin measurement	Percentage of patients 18-75 years of age with a diagnosis of diabetes with urine microalbumin measurement documented in the last 12 months	Gathering outside labs and records and updating the EHR Arranging urine microalbumin labs and physician visits when needed

The primary objective of the intervention was to have 50% of the patients meet all six measures (all or none compliance) by December 31, 2019.

To identify patients not meeting the quality goals, the RN ran a report using the EHR. Once the patients were identified, the RN used various strategies to engage patients to improve each quality measure (Table 1). On average, the RN spent ten hours per week working on these strategies.

In addition to engaging patients directly, the RN would meet with each physician for about 30 minutes per month to review their patients not meeting goals and to identify strategies to improve measurements. In some instances, this could be an adjustment in medication to better control glycemic levels or to meet BP measurement goals. When the physician requested an in-person follow-up with the patient, the RN would work with scheduling to arrange a visit.

Blood pressure management was defined by having the most recent blood pressure recorded less than 140/90. The RN worked with the rooming staff (licensed practical nurses and medical assistants) to ensure that the appropriate technique for evaluating blood pressure was being followed and would document in the EHR when a patient with diabetes was coming in and to ensure that before the patient left the clinic, they had a good blood pressure reading or a plan for follow-up if the blood pressure was not at goal.

Lastly, for patients who were actively smoking, the RN would provide education and smoking cessation resources, and if the patient was interested, they would assist with enrolling them in a smoking cessation course offered through the state of Florida or within the health system of the clinic. In some cases, the smoking history had not been updated accurately, and the RN would update this if needed and would engage rooming staff to ensure the smoking history was reviewed during the rooming process.

All statistical analyses were done using R version 4.0.3 (R Foundation for Statistical Computing, Vienna, Austria). This research received no specific grant from any funding agency in the public, commercial, or not-for-profit sectors.

## Results

One area that was identified during the RN’s review of these patients is that some patients had moved out of the local area, transitioned to another PCP, or changed insurance plans. These patients were removed from the PCP’s panel to accurately reflect the number of patients being cared for. Prior to this process being implemented, the clinic was dependent on patients notifying the office that they would no longer be receiving their care there. 

The results measured during the RN phase were compared to the previous year (June 1, 2018-March 31, 2019). One factor that was not considered at the beginning of this quality improvement project was the implementation of a new EHR. In June of 2018, the clinic was using Cerner EHR (Cerner Corporation). In October 2018, the clinic changed to an Epic EHR (Epic Systems Corporation). This made a notable impact on the quality measures between October 2018 and the RN intervention in April 2019 (pre-RN phase).

All-or-none compliance was found in 500 patients (40.0%) in the Cerner phase, 514 (24.6%) in the pre-RN phase, and 1549 (40.6%) in the RN phase. The pre-RN phase was associated with a lower all-or-none compliance compared to the Cerner phase, but there was no evidence of a difference in compliance in the RN phase compared to the Cerner phase (Table 2).

**Table 2:** All-or-none compliance (a) (changes in all-or-none compliance reflect documentation and reporting changes) OR: odds ratio; SE: standard error. ^a^ October 2018 was excluded from data analysis, as this was the month the new electronic health record (Epic) was implemented. ^b^ ORs and corresponding p-values for the association of phase with all-or-none compliance were estimated from a logistic regression model adjusting for physician. ^c^ The initial (intercept) and change (slope) in all-or-none compliance were estimated from linear regression models separately for each phase, where the dependent variable was the physician-level monthly compliance rate and month was the only predictor variable in the model. ^d^ Slope is significantly different from zero.

			Logistic regression		Linear regression	
Phase	Months	All or none compliance, No. (%)	Association with all or none compliance, OR (95% CI)^b^	p-value	Initial provider-level all or none compliance, % (SE)^c^	Change in provider-level all or none compliance per month, % (SE)^c^
Pre-Epic	06/2018-09/2018	500/1,249 (40.0%)	1.00 (reference)		39% (2.0%)	0.2% (1.1%)
Pre-Nurse	11/2018-03/2019	514/2,093 (24.6%)	0.48 (0.41-0.56)	< .001>	17% (2.4%)	3.8% (1.0%)^d^
Nurse	04/2019-12/2019	1,549/3,812 (40.6%)	1.01 (0.89-1.16)	.84	35% (1.6%)	1.2% (0.3%)^d^

**Figure 1:**
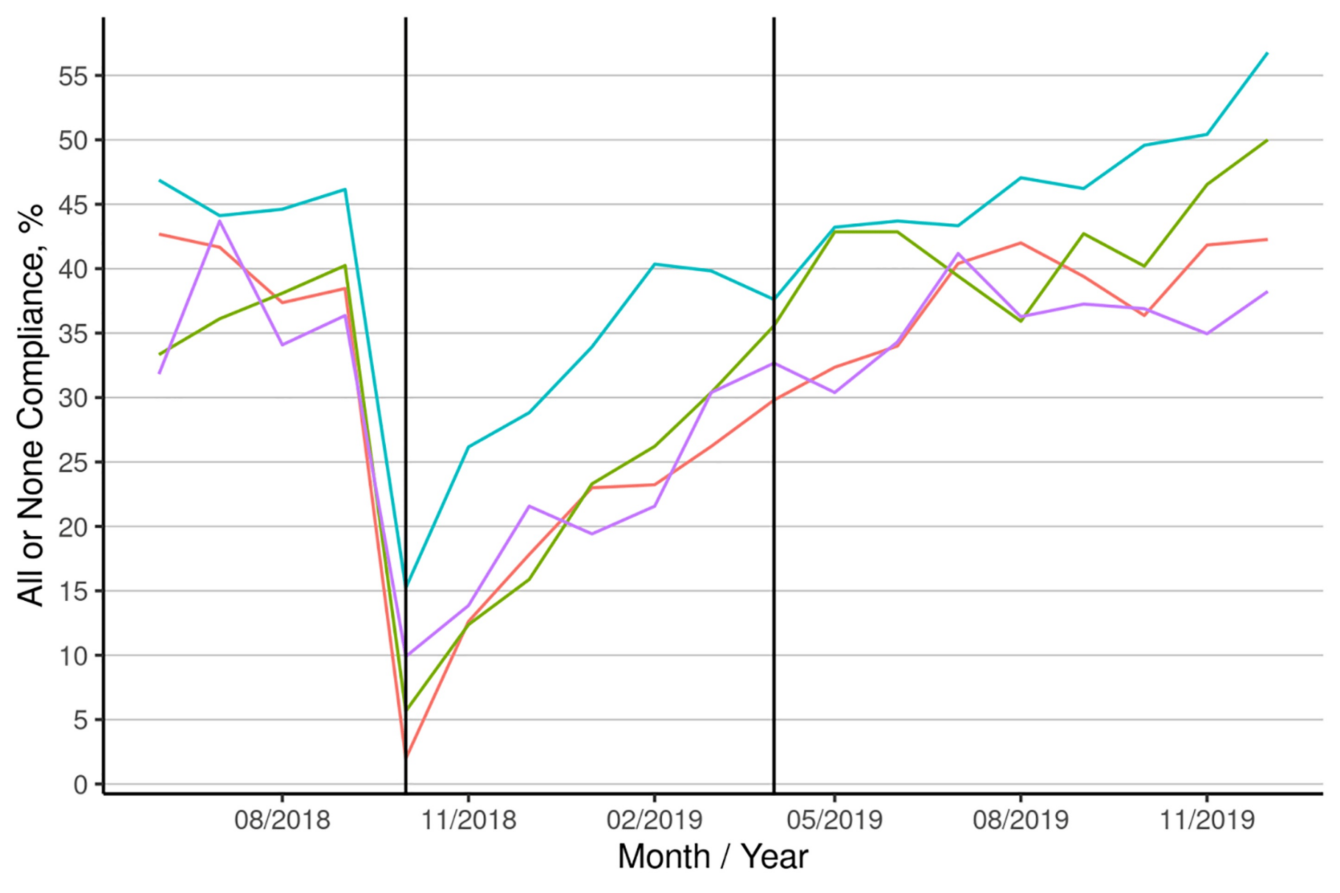
All-or-none compliance The three sections separated by vertical lines represent the three phases, left to right: pre-Epic, pre-nurse, and nurse. The colored lines represent the four providers’ all-or-none compliance rates

Figure 1 shows a sharp decline in all or none compliance with the implementation of Epic in October 2018.

Figure 1 illustrates the all-or-none compliance across three phases, pre-Epic, pre-nurse, and nurse, separated by vertical lines, with colored lines representing the compliance rates of the four providers over time. 

**Figure 2:**
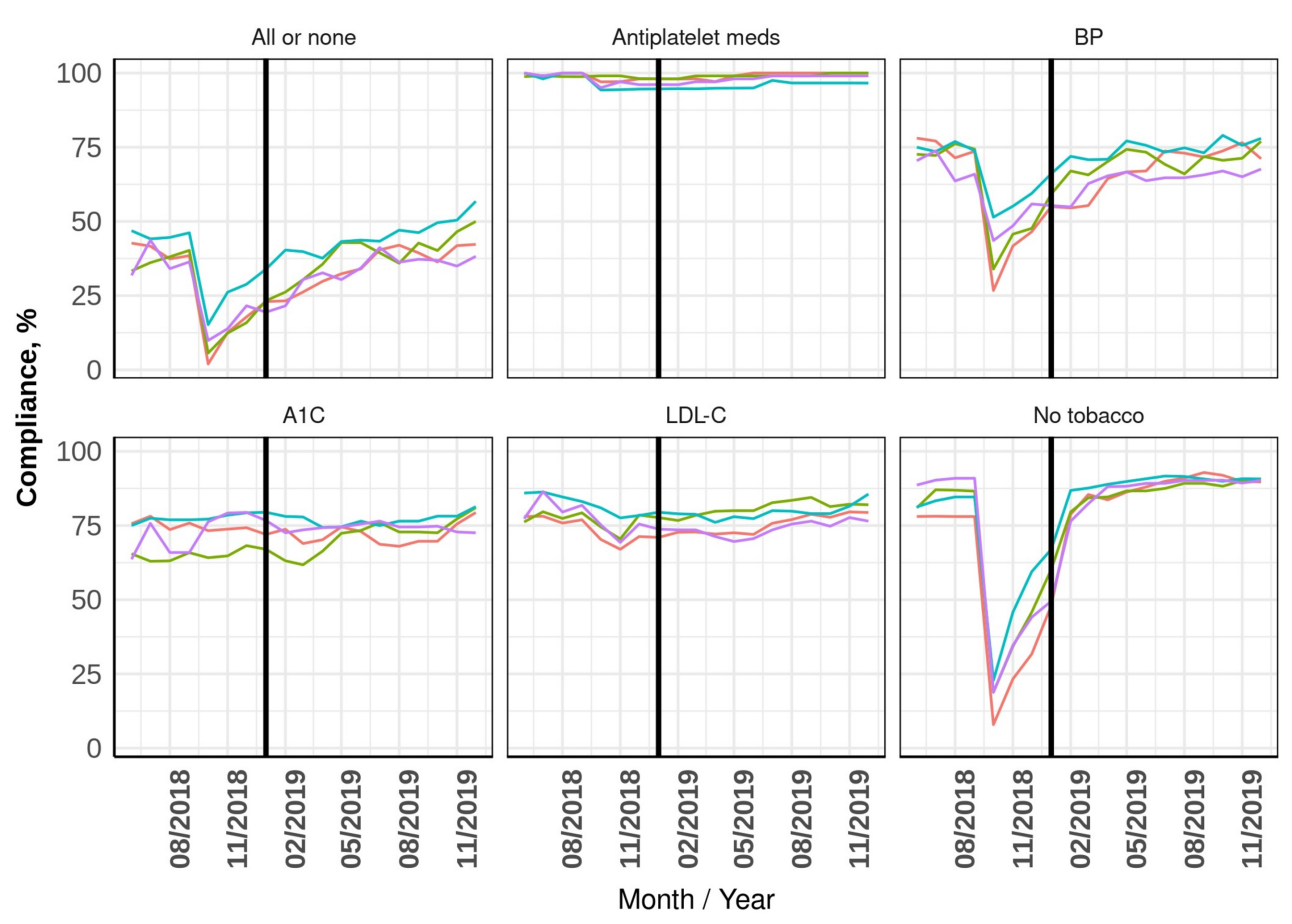
Percentage of patients meeting individual measures Conversion to the Epic electronic health record occurred in October 2018. Nurse intervention began in April 2019. The two sections separated by a vertical line represent pre-nurse and nurse phases, respectively. The colored lines represent the four providers’ compliance rates. BP indicates blood pressure; LDL-C: low-density lipoprotein cholesterol.

This initial decline was thought to be a result of delayed data transfer from the Cerner EHR to the Epic EHR, specifically tobacco use and blood pressure measurements. In November 2018, the mean compliance rate was 355.8 (17%) patients, and it increased by an average of 80 (3.8%) patients per month through the pre-RN phase (Table 2). This rate of improved compliance was attributed to the completion of data transfer to the Epic EHR, an improved method of tracking quality measures, and a user-friendly reporting system that provides real-time information on patients not meeting their quality goals. Figure 2 shows percentage of patients meeting individual measures.

In April 2019, when the RN intervention started, the mean compliance rate was 1334 (35%) patients, and it increased by an average of 46 (1.2%) patients per month until December 2019 (Table 2). The primary aim was to assess whether the RN intervention impacted all or no compliance rates; however, it is difficult to make any conclusions from this data due to the effects of the change in the EHR.

Nonetheless, the RN was instrumental in identifying criteria that were required by the EHR to satisfy certain quality measures. Most of these findings were unrelated to the physicians’ medical decision-making or quality of care delivered.

One of the technical challenges revealed was that medications that had not been electronically reconciled in more than 12 months were marked “expired” or “historical” medications in the EHR. As a result, they were reported as not meeting this quality measure. This was particularly challenging following the conversion to the new EHR because many of the medications were listed as historical and not active on the medication list. The RN identified these challenges and was instrumental in rectifying the erroneous reporting to accurately reflect the quality measure.

Another component of the EHR quality reporting identified by the RN was that if a patient’s tobacco use status had not been marked as reviewed in the past 12 months, the measure was marked as unmet. This was the case for both tobacco users and non-tobacco users. This measure was impacted the most by the change in the EHR (Figure 2); however, the RN was able to update records to accurately reflect tobacco status.

Several instances of high blood pressure readings occurred in other specialties outside of family medicine (e.g., oncology). Many of these cases would not have been identified if the RN were not reviewing the reports on a weekly basis. Blood pressure readings taken in the emergency department or in procedural areas were not included in the reporting. 

Furthermore, it was identified that some of the rooming staff were not using the appropriate technique for taking blood pressure readings in the clinic. As a result, the rooming staff were educated on the appropriate process for taking blood pressure and were instructed to wait 10 minutes and retake the measurement if the patient’s initial reading was high. Patient-reported blood pressure readings were excluded from the report.

The RN was able to identify individuals who were overdue for these measures and placed lab orders and arranged follow-up visits with the PCP. Most patients in the practice had their lab studies completed within the clinic, which was reported electronically in the EHR and satisfied the quality measure. 

Some patients opted to have lab studies done at external laboratories, which required manual entry into the EHR to satisfy the quality report. The RN was able to identify individuals who had already completed external labs ordered by the PCP or a physician outside of the practice, such as an endocrinologist. This resulted in an unanticipated impact on the RN’s dedicated time.

The RN also identified patients listed in the quality report who were no longer affiliated with the practice due to a change in insurance, a move out of the local area, or other undisclosed reasons. These individuals negatively impacted the quality reporting based on overdue labs, unreconciled medications, overdue tobacco use verification, and undocumented blood pressure measurements. Removing these individuals helped improve the accuracy of quality reporting.

Furthermore, the RN found patients in the report with diabetes erroneously listed in their medical problem list. In these situations, the PCP was asked to remove the diagnosis, if appropriate, to establish a more precise list of patients with diabetes within the practice.

## Discussion

Interpretation of this quality improvement project should consider that a new EHR was implemented during the study period, which had a greater-than-anticipated influence on documentation and reporting of diabetes quality measures. 

The CMS defines quality measures as “tools that help us measure or quantify health care processes, outcomes, patient perceptions, and organizational structure and/or systems that are associated with the ability to provide high-quality health care” [15]. Quality measures have become increasingly influential as health plans are using physician quality information for value-based contracting, pay-for-performance programs, provider networks, physician tiering, and more [16]. Furthermore, physician quality data have become easily accessible to the public through online programs, such as CMS Physician Compare [17]. As a result, quality data can be used to determine physician reimbursement and how patients choose their physicians.

Despite the growing demand to measure quality, PCPs find this burdensome, leading to physician burnout and lower quality of care [18,19]. Furthermore, there is concern that quality measurement has little impact on patient care, population health, and the reduction of health care costs. In a 2017 survey of family physicians, 62% cited a lack of evidence that meeting performance measures leads to better patient care [20]. Nonetheless, most physicians view quality care not only as a professional responsibility but also as the most important reason for their medical service [21].

The primary intent of this quality improvement project was to use nursing support to improve quality measures for 477 patients with diabetes in a family medicine clinic. The RN was asked to oversee six quality measures for these patients and was provided dedicated time (approximately 10 hours per week) to accomplish this task. 

The following sections outline the initiatives of the RN and some of the challenges faced during the implementation of this quality improvement project.

Medications

To ensure that patients with known cardiovascular disease were on anti-thrombotic (aspirin) therapy and that individuals meeting criteria for statin therapy were receiving the appropriate medication, the RN called and discussed the benefits of these medications with the patient. If the patient agreed to start aspirin therapy, the RN documented this in the medication list. If the patient agreed to statin therapy, a message was sent to the PCP to initiate therapy and arrange appropriate follow-up. If the patient had further questions, they were offered a telephone call from a clinical pharmacist or a follow-up visit with their PCP. If the patient reported an allergy, intolerance, or contraindication to these therapies, it was added to the allergy field in the EHR, which would report as meeting the goal.

Tobacco status

Individuals identified as using tobacco during the RN intervention phase were contacted by the RN and offered resources to assist with tobacco cessation. This included literature and assistance with enrollment in a free statewide assistance program. Appointments were made with the PCP if the patient had an interest in pharmacologic assistance with the cessation of tobacco use.

Blood pressure

When patients were identified as having their most recent blood pressure reading above the quality goal of 140/90, the RN arranged a visit with a member of the clinical team to reevaluate blood pressure and make medication adjustments if needed.

Laboratory

To meet the quality goal for blood sugar control, the patient needed a documented hemoglobin A1c of less than 8% within six months of reporting. To meet the goal for nephropathy screening, the patient needed a urine microalbumin report within 12 months of the measurement period.

Miscellaneous findings

It was hypothesized that providing RN support for patients with diabetes would improve documentation accuracy and process measure compliance within the EHR, with more than 50% of patients meeting all six recorded quality measures. It was anticipated that the RN would be able to engage patients and close gaps in care through education, assistance with overdue lab orders and office visits, and communicating with PCPs about patients needing closer follow-up or assistance. Although the RN did provide some level of direct patient engagement, most of the RN’s time was dedicated to addressing deficiencies in the EHR to adequately reflect quality goals already being met. It is not clear how much of this was a direct reflection of a new EHR, but many of these challenges were present prior to the conversion. As quality measurement continues to be a major emphasis in health care delivery, there is increasing pressure on physicians to ensure they are meeting these measurements. Along with addressing chronic and acute care needs, refilling medications, completing paperwork, and providing patient education, physicians need to be attuned to quality measures during routine office visits. Oftentimes, this is done by clicking buttons in the EHR to satisfy a measurement that has no influence on patient outcomes.

Although it was not the primary objective of the quality improvement project, this work helped to reveal how much influence EHR has on quality measures. Having a dedicated RN assisting with this work was key to addressing some of the deficiencies noted in the EHR. It is likely that most physicians would have little interest in satisfying an action in the EHR, such as clicking a button to verify tobacco use, if they know their patients are already meeting a quality measure. Satisfying the needs of the EHR can distract physicians from addressing the needs of the patient and can ultimately lead to a lower quality of care.

Other research studies have proven that a team-based approach to caring for patients with type 2 diabetes improves outcomes in glycemic control, hypertension, and lipid management [22]. The Complexities of Physician Supply and Demand: Projections From 2019 to 2034, a report released by the Association of American Medical Colleges, projects a shortage of between 17,800 and 48,000 primary care physicians by 2034 [23]. With the projected shortages in primary care physicians and the increasing number of people living with diabetes, it will be extremely important to utilize other members of the healthcare team to provide guidance and care for this patient population. 

In addition to RNs, pharmacists have been proven to play an important role in managing chronic conditions as part of the care team [24]. Healthcare teams that included both a pharmacist and an RN were able to reduce both blood pressure and blood glucose levels [22,24]. Pharmacists have the skillset to focus on medication adherence, side effects, dose adjustment, education, and contraindications to other prescriptions to assist primary care physicians in improving outcomes in chronic conditions [22,24,25].

Other studies have reported the benefit of incorporating diabetes educators into primary care practices. Grohmann et al. found that adding diabetes educators to primary care clinics improved patient experience by providing education and support to the primary care physicians [26]. Educators have more time to devote to key components of diabetes management, such as nutrition, lifestyle modification, and managing blood sugar with glucometers.

Although these additional resources can improve the total care of the patient living with diabetes, they may not be easy to incorporate into a primary care practice due to limited availability and limited funding. However, there are community-based programs that are available to partner with primary care practices and provide additional support to patients and physicians [22]. 

One of the limitations of this study was transitioning to a new EHR during the time of the study, which impacted the collection of quality measures. Assigning one RN to the various tasks and quality measures above, especially during the time of EHR transition, was met with unique limitations of its own, as previously mentioned. The time taken to review, reconcile charts, and counsel patients may also be a barrier, but highlights the importance of having an individual or individuals assigned to this task. Especially where the EHR itself can have limitations. In this study, we revealed the challenge in cross-communication between EHR systems at one site. This challenge would be expected to be more so if obtaining information across different EHRs at different sites, as it is constantly updating, depending on a patient’s encounters from various institutions, whose outside records may or may not be available.

## Conclusions

Delivering quality care to patients is a team approach, and leveraging members of the health care team beyond the physician can allow greater focus on patient care and quality. As EHRs move beyond simple electronic record keeping into complex data collection and reporting systems that allow users to track and measure outcomes, including quality, it is important to understand their deficiencies. As this quality improvement project demonstrates, it is essential that healthcare teams dedicate staff to oversee quality data, commit to understanding how an EHR captures and reports data, and, most importantly, ensure that the needs of the patient are truly being addressed. With increased transparency on quality scores and reimbursement being closely tied to quality measures, this cannot be left solely up to the physicians, especially when the work is being done but not adequately reported. Further research should evaluate whether improvements in quality reporting translate into measurable improvements in patient-level diabetes outcomes.
